# Decorin: A Growth Factor Antagonist for Tumor Growth Inhibition

**DOI:** 10.1155/2015/654765

**Published:** 2015-11-30

**Authors:** Tero A. H. Järvinen, Stuart Prince

**Affiliations:** ^1^School of Medicine, University of Tampere, 33014 Tampere, Finland; ^2^Department of Orthopedics & Traumatology, Tampere University Hospital, 33521 Tampere, Finland

## Abstract

Decorin (DCN) is the best characterized member of the extracellular small leucine-rich proteoglycan family present in connective tissues, typically in association with or “decorating” collagen fibrils. It has substantial interest to clinical medicine owing to its antifibrotic, anti-inflammatory, and anticancer effects. Studies on DCN knockout mice have established that a lack of DCN is permissive for tumor development and it is regarded as a tumor suppressor gene. A reduced expression or a total disappearance of DCN has been reported to take place in various forms of human cancers during tumor progression. Furthermore, when used as a therapeutic molecule, DCN has been shown to inhibit tumor progression and metastases in experimental cancer models. DCN affects the biology of various types of cancer by targeting a number of crucial signaling molecules involved in cell growth, survival, metastasis, and angiogenesis. The active sites for the neutralization of different growth factors all reside in different parts of the DCN molecule. An emerging concept that multiple proteases, especially those produced by inflammatory cells, are capable of cleaving DCN suggests that native DCN could be inactivated in a number of pathological inflammatory conditions. In this paper, we review the role of DCN in cancer.

## 1. Introduction

Decorin (DCN) is the best characterized member of the small leucine-rich proteoglycan (SLRP) family of extracellular matrix (ECM) proteins. Due to its close interactions with collagen fibers in the ECM—that is, DCN “decorates” collagen fibers—the proteoglycan was named decorin early on [1]. DCN was initially cloned in 1986 and thought at the time to be a structural constituent of the ECM [1]. However, soon it was established that DCN had a role beyond just a structural component of the ECM, as it became evident that it influenced cellular functions such as proliferation, spreading, migration, and differentiation, as well as being a physiological regulator of inflammation [2–5]. Some of these early findings were derived from tumor cells [2–5], where it was shown that DCN inhibited cancer cell proliferation and spreading. These studies sparked a two-decade-long quest that established DCN as a promising antitumor agent to treat human cancer patients [6].

**Figure 1:**
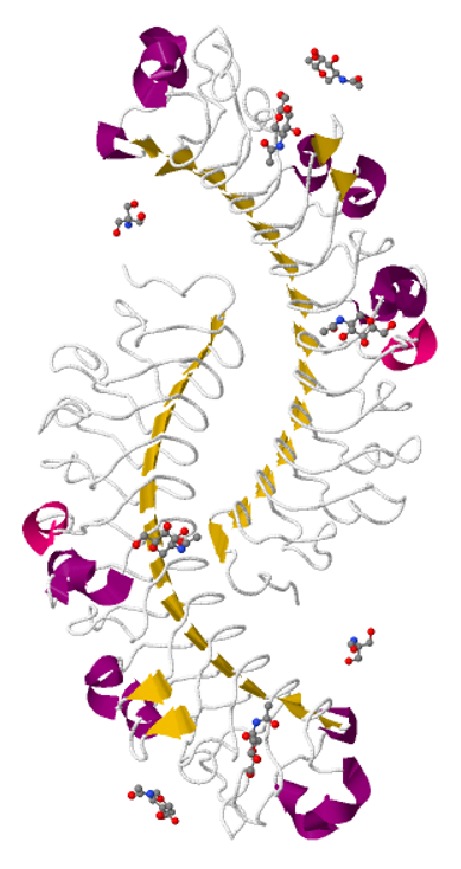
*Structure of decorin*: mammalian decorin (DCN) contains a monomeric protein core of 42 kDa and a single chondroitin/dermatan sulfate glycosaminoglycan (GAG) chain. DCN exists as a dimer in physiological solutions and is the best characterized member of the growing family of SLRPs. Structurally, it has a domain of tandem leucine-rich repeats (LRRs, altogether 12 LRRs), flanked on both sides by two cysteine-rich regions. Decorin dimer structure (from PDB 1XKU). Images prepared with JMOL program. The N-terminus is in the “middle” of the antiparallel homodimer.

**Figure 2:**
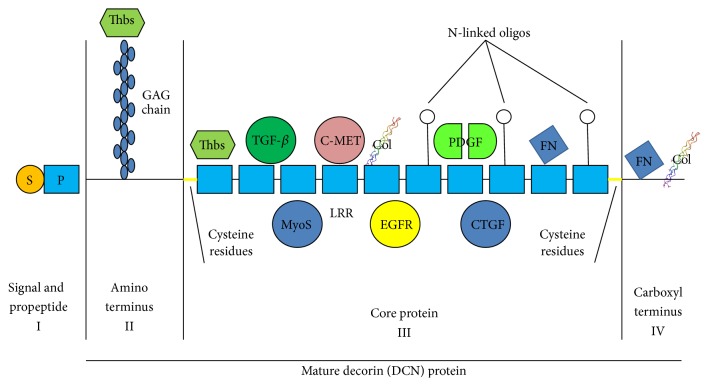
Decorin interacts with multiple growth factor signaling pathways crucial for cancer growth. Schematic drawing of the molecular structure of decorin (DCN). All four domains, I–IV, of decorin core protein are indicated. DCN has a monomeric protein core and a single chondroitin/dermatan sulfate glycosaminoglycan (GAG) chain. Structurally, it has a domain of tandem leucine-rich repeats (LRRs), flanked on both sides by two cysteine-rich regions. DCN interacts with a wide set of different signaling molecules; among them are different isoforms of transforming growth factor-*β* (TGF-*β*), platelet-derived growth factor (PDGF), epidermal growth factor receptor (EGFR), and ErbB1–4 receptor tyrosine kinases, myostatin (MyoS), connective tissue growth factor/CCN2 (CTGF), thrombospondin (Thbs), collagen (Col), and fibronectin (FN), implicated in cancer progression. The active/binding sites of DCN for TGF-*β*, CCN2, c-Met, and EGFR neutralization/binding all reside in different parts of the DCN molecule. Thus, in theory, a single DCN molecule could simultaneously sequester multiple important mediators of tumor growth and antagonize multiple signaling pathways crucial for tumor growth and progression. Thus, owing to this multifunctionality, DCN may exert its anticancer effects through multiple molecular approaches that all contribute to varying degree to its biological effects on cancer cells and tumor environment.

Mammalian DCN contains a monomeric protein core of 42 kDa and a single chondroitin/dermatan sulfate glycosaminoglycan (GAG) chain, attached to a serine residue near the N terminus [1, 7] (Figures 1 and 2). DCN exists as a dimer in physiological solutions [8, 9] and as a monomer when bound to collagen [10] (Figures 1 and 2) and is the best characterized member of the growing family of SLRPs [8, 9]. Structurally, it has a domain of tandem leucine-rich repeats (LRRs, altogether 12 LRRs), flanked on both sides by two cysteine-rich regions [8, 9] (Figures 1 and 2). SLRPs have been grouped into three different classes on the basis of gene organization, amino acid sequence similarity, number of LRRs, and the spacing of cysteine residues in the N-terminal segment [8]. DCN belongs to class I SLRPs with biglycan (BGN) and asporin [8, 11]. The structural similarities between different SLRPs provide an explanation as to why they share some of their biological functions [11–13]. DCN has been implicated to play a role in the development and progression of cancer and a substantial amount of work has been published on its therapeutic anticancer effects. We will review the role of DCN in cancer development and highlight its vast therapeutic anticancer potential in this review article.

## 2. Loss of DCN Leads to Spontaneous Tumor Development

DCN knock-out (KO) mice are fertile and show no obvious malformations in their tissues [14]. Their skin and tendons are mechanically fragile owing to the DCN function of regulating collagen fibrillogenesis [14]. When a double KO of DCN and another closely related SLRP, BGN, was generated, the phenotype in the double-KO mice was more severe than in the DCN KO mice [12]. The DCN-BGN double-KO phenotype is reminiscent of a specific subtype of Ehlers-Danlos syndrome (EDS), the progeroid variant, a clinically and genetically heterogeneous connective tissue disorder characterized by skin hyperextensibility, joint hypermobility, and tissue fragility [12, 15]. Taken altogether, the presence of BGN compensates for the loss of DCN in the DCN KO. In this context, it is worth noting that asporin shares the collagen binding site with DCN [11]. Theoretically, asporin could compensate for both DCN and BGN in double-KO animal and alleviate the phenotype caused by the absence of its two family members.

Despite the compensatory effect of BGN (and the potential compensatory role of asporin) in the absence of DCN, 30% of DCN KO mice developed spontaneous intestinal tumors and a high-risk diet enriched in fat amplified and accelerated the tumor development and growth initiated by DCN deficiency [16]. Interestingly, E-cadherin, a protein that regulates cell-cell adhesion, epithelial-mesenchymal transition, and metastasis, was almost completely lost from the DCN KO intestine, and loss of DCN and E-cadherin accelerated colon cancer cell growth and invasion [16]. DCN and p53 tumor suppressor double-KO mice, in turn, have a significantly faster rate of lymphoma development than p53 KO alone and succumb to thymic lymphomas months earlier than p53 KO mice [17]. Most recently, the absence of DCN in KO mice has been shown to promote chemically induced hepatic carcinogenesis [18, 19]. Genetic ablation of DCN led to enhanced tumor occurrence as compared to wild-type animals in different models of hepatic cancer [18, 19]. Taken together, all of the data generated on DCN KO mice suggest that DCN is a potent tumor suppressor gene [6, 16–19].

There have not been any mutations reported for DCN in human malignancies, whereas such mutations cause human disease called congenital stromal corneal dystrophy (CSCD), the disease characterized by corneal opacities and vision impairment [20].

## 3. Reduced Expression of DCN in Human Tumors

A large number of publications from different types of human cancers have shown that DCN expression in tumors is significantly reduced from the levels expressed in normal tissues or very often totally lost from tumor tissue [21, 22]. The reduced or total absence of DCN expression has been reported to take place in breast, colon, prostate, vascular, and bladder cancers, liposarcomas, myelomas, and malignant peripherial nerve sheath tumors [21–26]. In line with these findings, there is also evidence from human cancers that DCN expression decreases with the malignant transformation of tumor cells, its expression being lost in the transformation from benign to malignant tumors or its expression being lowest or totally absent in the most aggressive tumors [21–25, 27]. In support of this concept, low levels of DCN in cancers are associated with significantly poorer outcome and a shorter time to progression than with patients expressing higher levels of DCN in breast, lung, and soft tissue cancers as well as in myeloma [21–23, 28]. The reduced expression of DCN is not restricted only to tumor cells in cancer progression; stromal expression of DCN is also decreased by soluble factors secreted by tumor cells [29–31]. Myeloma cells secrete CCL3 chemokine that suppresses DCN expression in the surrounding bone marrow stroma [31] because stromal/osteoblast derived DCN is known to inhibit myeloma cell proliferation and survival [29, 31, 32]. Breast cancer cells, in turn, secrete periostin that sequesters DCN and by doing so the cancer cells provide themselves an opportunity to grow invasively and migrate without inhibitory activity from DCN [30].

Very recently, the reduced DCN expression has been linked to osteosarcoma development in Li-Fraumeni syndrome (LFS) [33]. It was shown that the LFS-cells exhibit impaired expression of the imprinted gene H19 [33]. Restoration of H19 expression in LFS-cells facilitated normal cell differentiation and repressed tumorigenic potential. It was found that H19 mediates suppression of LFS-associated osteosarcoma formation through DCN [33].

## 4. DCN Sequesters Transforming Growth Factor-***β***

Transforming growth factor-*β* (TGF-*β*) was the first growth factor DCN was identified to interact with [5] (Figure 2), and it became evident that DCN effectively inhibits TGF-*β* induced cancer cell spreading and proliferation in different cancer cell lines [5]. Later it was shown in different animal models that DCN is very capable of reducing tissue fibrosis and inflammation caused by TGF-*β* [4, 34–38]. The DCN core protein binds to all isoforms of TGF-*β*, namely, TGF-*β*1, TGF-*β*2,  and TGF-*β*3 (Figure 2), although the chondroitin sulfate GAG side chain of DCN slightly interferes with the core protein's binding of TGF-*β* [39]. Thus, DCN traps TGF-*β* in the ECM before it can bind to its receptors on the cell surface [36]. In addition to neutralizing all isoforms of TGF-*β*, DCN also binds and neutralizes another member of the TGF-*β* superfamily capable of inducing fibrosis and restricting tissue regeneration, myostatin [37, 40] (Figure 2). In the case of myostatin inhibition, DCN sequestration shuts down myostatin's growth inhibitory effects on myofibers and thus DCN stimulates skeletal muscle regeneration [37, 40, 41]. Due to its potency in reducing inflammation and fibrosis, DCN has been proposed to be a physiological TGF-*β* inhibitor that limits the duration of TGF-*β* responses in inflammation and tissue repair [4, 36]. This claim is supported by the pro-inflammatory phenotype identified in DCN KO in different fibrotic disease models as well as the inflammation suppressive effects of exogenous DCN supplemented in experimental treatment trials [4, 34, 36, 42, 43].

## 5. DCN: A Pan-Receptor Tyrosine Kinase Inhibitor

ErbB receptor tyrosine kinases (RTKs) are heavily involved in the growth of several common human carcinomas [44, 45]. These receptors are amplified and overexpressed in a large number of tumors, most notably in breast cancer, where the ErbB2 gene at 17q12 is known to be amplified in 20% of the carcinomas and known to drive cancer progression [44, 45]. DCN binds directly to the epidermal growth factor receptor (EGFR/ErbB1) and inhibits its activity as well as the activity of another member of the ErbB RTKs, namely, ErbBs2–4 [46–50] (Figure 2). After binding DCN, the ErbB receptors dimerize and subsequently undergo caveolin-mediated internalization and degradation [50].

DCN is a pan-RTK inhibitor as it also inhibits other RTKs outside of the ErbB family. It interacts with c-Met, hepatocyte growth factor (HGF) receptor tyrosine kinase (Figure 2). By binding to the c-Met receptor DCN induces a short activation, which is then followed by a rapid inactivation of the receptor by intracellular degradation [51, 52]. DCN also binds and inhibits the biological function of vascular endothelial growth factor receptor 2 (VEGFR2) (please see Section 7 for more details) and insulin-like growth factor-1 receptor (IGF-IR) [53, 54].

## 6. Other Signaling Molecules Affected by DCN

In addition to TGF-*β* and the RTKs, DCN also interacts with a wide set of different signaling molecules implicated in cancer progression. In an analogous antagonism to TGF-*β*, DCN sequesters platelet-derived growth factor (PDGF) before it can bind to its receptors on the surface of target cells [55] (Figure 2). The DCN-provoked inhibition on the PDGF-dependent phosphorylation of the PDGF receptor results in the attenuation of cancer cell migration [19, 55]. DCN also binds and neutralizes molecules such as connective tissue growth factor (CTGF/CCN2), low-density lipoprotein receptor-related protein 1 (LRP-1), thrombospondin (THBS), and Wnt-1-induced secreted protein 1 (WISP) [56–59] (Figure 2). All of these molecules have been directly shown to enhance cancer growth and progression in different cancer models and human cancers [56–59]. The ECM protein periostin, which is abundantly expressed by a large number of different human cancers, binds and neutralizes DCN [30].

The active/binding sites of DCN for TGF-*β*, CCN2, c-Met and EGFR neutralization/binding all reside in different parts of the DCN molecule [57, 59] (Figure 2). Thus, in theory, a single DCN molecule could simultaneously sequester multiple important mediators of tumor growth and antagonize multiple signaling pathways crucial for tumor growth and progression [36] (Figure 2). Thus, owing to this multifunctionality, DCN may exert its anticancer effects through multiple molecular approaches that all contribute to varying degree to its biological effects on cancer cells and tumor environment [6] (Figure 2).

## 7. DCN in Angiogenesis

Tumors induce thegrowth of new blood vessels from preexisting ones. This process, angiogenesis, is a vital requirement for tumor growth because the formation of new blood vessels allows a variety of mediators, nutrients, and oxygen to reach the rapidly growing tumor cells [60, 61]. DCN has been implicated to be involved in the regulation of angiogenesis with conflicting outcomes. In terms of cancer, DCN has been shown to have an antiangiogenic effect on tumor angiogenesis [62]. Among the RTKs DCN inhibits, VEGFR2 inhibition is the most significant for DCN induced inhibition of tumor angiogenesis [54]. DCN binds directly to the ectodomain of VEGFR2 at a site that partially overlaps with the canonical binding site for VEGF-A [54]. DCN has been shown to inhibit tumor cell-mediated production of the angiogenic molecules vascular endothelial growth factor A (VEGF-A), hypoxia inducible factor-1*α* (HIF-1*α*), and c-Met, whilst simultaneously inducing the production of the antiangiogenic, angiostatic molecules thrombospondin-1 and tissue inhibitor of metalloproteinases 3 (TIMP3) [63, 64]. In addition to influencing the balance of anti- and proangiogenic factors, the antiangiogenic mechanisms of DCN in tumor angiogenesis involve autophagy [62]. Namely, DCN induces the expression of paternally expressed gene 3 (Peg3), an imprinted tumor suppressor gene, and Peg3 relocates into autophagosomes [65]. DCN evokes Peg3-dependent autophagy in both microvascular and macrovascular endothelial cells leading to suppression of angiogenesis [65].

On the other hand, there is also evidence for DCN supporting angiogenesis outside of tumor angiogenesis [66, 67]. DCN has been shown to play a proangiogenic role by supporting endothelial cell adhesion to type I collagen (Figure 2) and to *α*1*β*2-integrin and thus promoting the integrin-collagen interaction [68]. DCN deficiency, in turn, leads to impaired angiogenesis in the injured cornea [66], while a decorin mimic supports endothelial cell proliferation and migration [69]. Furthermore, DCN was also identified as an angiocrine (endothelial cell derived growth factor for organ-specific tissue regeneration) factor for endothelial cell-driven liver regeneration [70]. Thus, depending on the cellular and molecular microenvironment where angiogenesis occurs, DCN can exhibit either a proangiogenic or an antiangiogenic activity [71]. Nevertheless, DCN exhibits exclusively antiangiogenic activity in tumorigenesis-associated angiogenesis and in various inflammatory processes [70, 71].

## 8. DCN as a Therapeutic Anticancer Drug In Vivo

Vast amounts of scientific data have shown that the administration of DCN can inhibit tumor growth and progression in vivo. Early studies utilizing virus-mediated gene therapy showed DCN transduced into tumor cells inhibited the growth of lung, colon, and squamous cell carcinomas in vivo [72]. DCN expression by osteosarcoma cells, in turn, inhibited their capability to send distant metastases to the lungs [73]. Similarly, virus-delivered DCN slowed the growth of breast cancer and prevented its distant spreading, that is, metastasis to various organs [74–76]. The expression of DCN by virus-mediated gene therapy in an experimental glioma model prolonged survival and inhibited tumor growth [77, 78]. The size of the tumors was directly proportional to the timing of the DCN gene transfer as well as to the expression levels of DCN attained by the gene transfer [77]. The tumor growth inhibitory activity of DCN gene therapy has also been shown in prostate and pancreatic cancer models [79, 80].

In addition to in vivo gene therapy studies, where DCN has been expressed from within virus-infected cells, in vivo tumor treatment studies with a recombinant DCN core protein have been carried out with considerable success. Treatment of A431 squamous cell carcinoma and breast carcinomas transfected with DCN cDNA resulted in tumor cell apoptosis, reduced EGFR signaling, and retarded tumor growth [46, 49, 81]. Systemic administration of DCN protein to treat breast cancer inhibited tumor growth effectively and reduced metastatic spreading of the tumor cells [52, 82].

## 9. Inactivation of DCN by Protease Mediated Cleavage in Human Pathologies

The multifunctional roles of DCN are due to its capacity to modulate the activity of a wide variety of proteins, such as growth factors or their cell surface receptors, and structural matrix proteins, via direct binding. Impairment of DCN binding to its partners due to aberrant DCN degradation is linked to fibrotic diseases and fibrotic wound healing. Several proteases as well as growth factors are known to be capable of cleaving DCN making it inactive against some of the growth factors or collagen it is capable of binding to. Amongst different proteases, DCN has been shown to be cleaved by matrix metalloproteinases-2 (MMP-2), MMP-3, MMP-7, membrane type 1-matrix metalloproteinase (MT1-MMP), cathepsin D, ADAMST-4 (adamalysin with thrombospondin type 1 motifs), ADAMST-5, and interleukin-1*β* [83–86]. Furthermore, inflammatory cells produce proteases capable of cleaving DCN. Cytotoxic lymphocytes produce granzyme B, a serine protease, while neutrophils produce neutrophil elastase, both of which are capable of cleaving DCN [87–90]. The cleavage of DCN by proteases derived from inflammatory cells is of special significance, because recent evidence suggests that DCN fragments can function as proinflammatory signaling molecules, so-called damage-associated molecular patterns (DAMPs), capable of inducing sterile inflammation [91]. DAMPs are recognized by pattern recognition receptors (PRRs), such as Toll-like receptors (TLR), and can trigger an inflammatory response [91]. Whether the DCN-induced sterile inflammation has any clinical significance is a relevant question, as an overwhelming amount of evidence points to DCN having a substantial anti-inflammatory effect in inflammatory, fibrotic diseases; phenotype in the absence of DCN (KO) is always both proinflammatory and profibrotic, whereas the in vivo treatment trials with exogenous DCN exclusively report both anti-inflammatory as well as antifibrotic effects [2, 4, 34, 36–38, 92].

Inactive DCN fragments generated by protease cleavage as well as the general reduced expression of DCN have been linked to human pathologies [93]. Namely, catabolic fragments of human DCN have been identified from scars and fibrotic diseases, and the impairment of DCN binding to its partners due to aberrant DCN degradation has been directly linked to fibrotic wound healing [94, 95]. Recently, it was shown that UV light exposure on skin can induce the expression of granzyme B, which cleaves DCN and leads to the appearance of wrinkles and the loss of normal collagen density in the skin [87]. In a similar fashion, it has been shown that DCN cleavage by granzyme B leads to aneurysm ruptures and subsequent deaths of the animals in an experimental aortal aneurysm model [88]. Furthermore, granzyme B can activate TGF-*β* [87]. In other words, the simultaneous cleavage of antifibrotic DCN and the activation of profibrotic TGF-*β* by granzyme B turn on a “profibrotic program” in inflammatory situations.

Furthermore, it has been shown that the majority of DCN exists in a cleaved, inactive form called “decorunt” in aged human skin, whereas most of the DCN in young human skin is in its full size [94, 95]. As DCN is involved in the regulation of collagen fibrillogenesis, it has been postulated that the loss of skin extensibility and fragility associated with aging is related to DCN being cleaved into inactive “decorunt” fragments [94, 95]. Interestingly, none of the DCN cleavage sites reported for different proteases generate the DCN fragments identified in “decorunt” [94, 95]. Thus, one can postulate on the existence of as yet unidentified protease(s) capable of cleaving and inactivating DCN [94, 95].

## 10. Limitations of DCN

Despite the vast amount of positive anticancer and antifibrotic results obtained with DCN treatment in various animal models, lack of any detectable toxicity, and extensive preclinical work conducted on it, DCN has not reached the clinic as a drug. There might be several reasons for that, such as an inadequate half-life in the circulation and the need for high dosing, but another reason is the fact that DCN is hard to mass-produce in a fashion that meets the regulatory criteria for human drugs. Namely, DCN is a proteoglycan, and the heterogeneity of its single GAG chain makes recombinant DCN produced in mammalian cells heterogeneous in size and composition [5]. The GAG chain is not needed for the antifibrotic activity of DCN [39], most of the anticancer effects of DCN have been generated with recombinant DCN with no GAG attached to it, and most of the interactions DCN has with growth factors or their receptors are through direct binding to the DCN core protein. The GAG is attached to a serine residue at position 4 of DCN [1], and it is possible to produce recombinant DCN without GAG attached to it by simply mutating the serine residue critical for the GAG attachment [7, 97].

## 11. Recombinant DCN Variant with Enhanced Biological Activity

**Figure 3:**
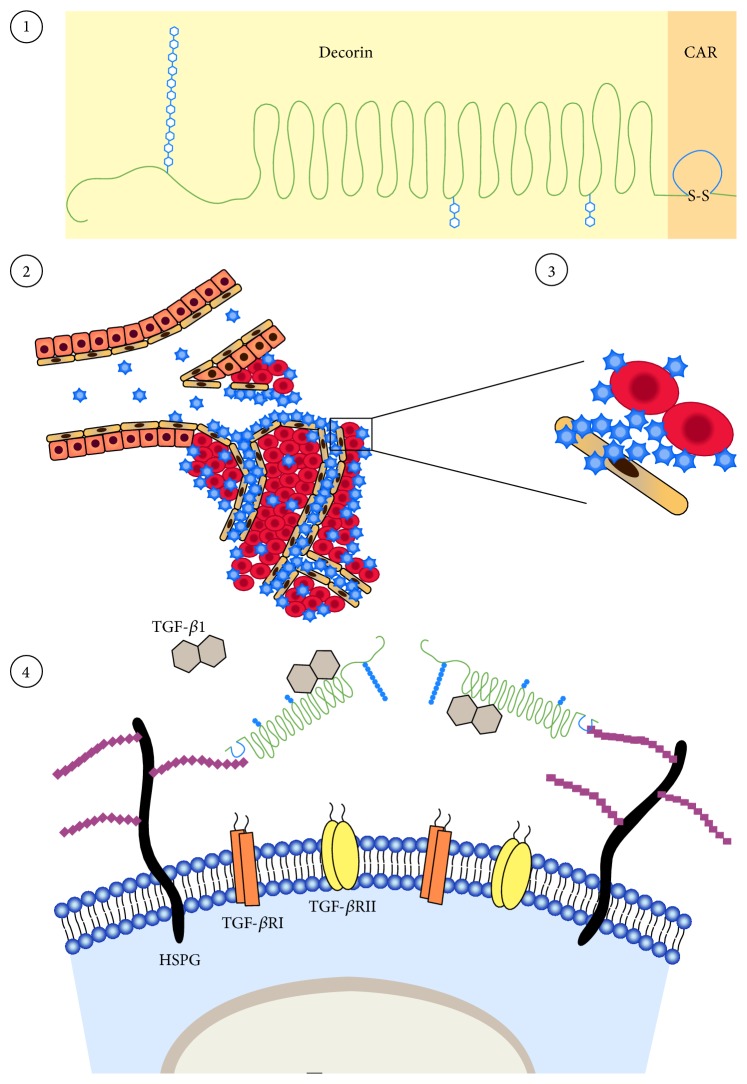
Schematic representation of the mechanism of action of the multifunctional therapeutic molecule CAR-decorin. CAR-decorin ① is a systemically administered, target-seeking, multifunctional biotherapeutic that inhibits numerous growth factors involved in tumor growth and progression. The molecule can be targeted to the angiogenic vasculature, whether it is induced by injury or by rapid cancer growth, taking place at any organ of the body ② (or multiple organs, i.e., metastases, simultaneously). The CAR homing peptide targets angiogenic vasculature ② and as it is a potent cell and tissue penetrating peptide, it can penetrate deep into target organ ③. Thus, the peptide (and any payload attached to it) then extravasates into surrounding tissue ③, where it binds to its receptor(s) on the cell surface of the target cells ③. CAR binding to heparan sulfate proteoglycans provides docking sites in the proximity of such growth factors as TGF-*β*1 and TGF-*β*2 ④, facilitating the neutralization of these growth factors by the therapeutic part of the molecule, decorin ④. This mechanism results in a therapeutic response. Picture by Helena Schmidt; reproduced with permission from Finnish Medical Journal Duodecim (originally published in [96]).

As the manufacturing issues of DCN can be solved fairly easily by a simple site-directed mutagenesis [7], further enhancement of its biological activity could make it an even more appealing drug candidate to pursue as a human therapeutic drug. We have recently attained that goal by developing a systemically administered, targeted, for example, inflammation- and angiogenesis-homing version of DCN core protein [34, 36, 98] (Figure 3). The angiogenesis- and inflammation-specificity of our enhanced DCN core protein is obtained by fusion to a small peptide that functions as an address tag and delivers systemically administered DCN to angiogenic and inflammatory vasculature [34, 36] (Figure 3). This small peptide, dubbed “CAR” (its sequence being CARSKNKDC) homes specifically to angiogenic blood vessels forming in tumors and regenerating tissues (such as wounds) [99, 100] and can deliver increased amounts of DCN in a tissue-specific manner with a significant therapeutic advantage over ordinary DCN core protein [34] (Figure 3). Furthermore, the fusion of CAR to recombinant DCN further enhances its neutralization of TGF-*β* stimulated cancer cell proliferation and spreading significantly [34] (Figure 3). The molecular explanation for the enhanced biological activity of CAR-DCN is that the CAR peptide binds to heparan sulfate proteoglycans (HSPGs) on cells [34, 99]. TGF-*β*1 and TGF-*β*2, in turn, also bind heparan sulfate and HSPG binding increases their biological activity (Figure 3). Thus, CAR mediated binding of CAR-DCN to HSPGs may enhance the neutralizing effect of the fusion protein by bringing it into the proximity of the HSPG-binding TGF-*β*s [34, 36, 97] (Figure 3). The CAR peptide has also been recently shown to target inflammatory vasculature, but not normal vasculature, in diseases affecting lungs [100–105] and to deliver different pharmaceutical agents (even large nanoparticle conjugates containing drugs) in target organ-specific fashion to diseased lungs [101–105]. Furthermore, CAR peptide has been used successfully to target mesenchymal stem cells to infarcted myocardium [106, 107]. Thus, CAR-targeted DCN could also be useful in the treatment of other conditions outside of cancer and healing wounds in which nontargeted DCN has shown activity and where there is angiogenesis or inflammation affecting nearby vasculature (Figure 3).

## 12. Conclusion

DCN is a well-studied member of the extracellular small leucine-rich proteoglycan family present in a variety of tissues and has substantial interest to clinical medicine owing to its antifibrotic and anticancer effects. Studies on DCN knockout mice have established that a lack of DCN is permissive for tumor development and it is regarded as a tumor suppressor gene. A reduced expression or a total disappearance of DCN has been reported to take place in various forms of human cancers during tumor progression. Furthermore, when used as a therapeutic molecule, DCN has been shown to inhibit tumor progression and metastasis in experimental cancer models. DCN affects the biology of various types of cancer by directly or indirectly targeting large numbers of crucial signaling molecules involved in cell growth, survival, metastasis, autophagy, and angiogenesis. The active sites for the neutralization/binding of different growth factors all reside in different parts of the DCN molecule. Multiple proteases, especially those produced by inflammatory cells, are capable of cleaving and inactivating DCN, indicating that DCN could be inactive in a number of pathological diseases involving an inflammatory component. Thus, the effective application of DCN core protein in human medicine will require strategies to deliver large amounts of the intact and functional protein specifically to where it is most needed, as well as other possible enhancement strategies. The fusion of DCN core protein together with the angiogenesis- and inflammation-homing peptide “CAR” is a significant step in this direction.
